# Allelic variants of the Melanocortin 4 receptor (*MC4R*) gene in a South African study group

**DOI:** 10.1002/mgg3.180

**Published:** 2015-10-23

**Authors:** Murray Logan, Maria‐Teresa Van der Merwe, Tyren M. Dodgen, Renier Myburgh, Arinda Eloff, Marco Alessandrini, Michael S. Pepper

**Affiliations:** ^1^ Department of ImmunologyUniversity of PretoriaPretoriaSouth Africa; ^2^ Faculty of Health SciencesInstitute for Cellular and Molecular MedicineUniversity of PretoriaPretoriaSouth Africa; ^3^ Department of EndocrinologyUniversity of PretoriaPretoriaSouth Africa; ^4^ Department of PharmacologyUniversity of PretoriaPretoriaSouth Africa; ^5^ Department of Genetic Medicine and DevelopmentFaculty of MedicineUniversity of GenevaGenevaSwitzerland

**Keywords:** Genotype–phenotype correlation, melanocortin 4 receptor, obesity, South Africa

## Abstract

Obesity is a global epidemic that results in significant morbidity and mortality. Mutations in the melanocortin 4 receptor (*MC4R*) gene, which codes for a G‐protein‐coupled receptor responsible for postprandial satiety signaling, have been associated with monogenic obesity. The prevalence of obesity is on the increase in South Africa, and it is hypothesized that mutations in *MC4R* are a contributing factor. The aim of this study was to perform a retrospective assessment of the relationship between allelic variants of *MC4R* and BMI in a South African study cohort. DNA was isolated from a demographically representative cohort of 297 individuals and the entire *MC4R* gene sequenced by Sanger sequencing. Eight previously reported *MC4R* variants were identified in 42 of the 297 (14.1%) study participants. The most frequently observed *MC4R* alleles were V103I (4.0%), I170V (1.5%), and I198I (1.2%), while the remaining five variants together constituted 1.18%. Five compound heterozygotes were also detected. Although *MC4R* variants were rare, the majority of variation was observed in individuals of Black African ancestry. No statistically significant associations with BMI were reported. Given that lifestyle interventions have limited success in decreasing obesity, there is an urgent need to perform large‐scale population studies to further elucidate the molecular underpinnings of this disease.

## Introduction

Obesity (body mass index [BMI] >30 kg/m^2^) is recognized as a chronic disease by the World Health Organization (WHO). It has reached pandemic proportions (Swinburn et al. 2011) and the prevalence continues to increase in the majority of African countries, particularly in individuals living in urban areas. In several parts of South Africa, the prevalence of combined overweight and obesity (BMI > 25) in economically active (18–65 years) Black African women has reached the alarming figure of 75% (Van der Merwe and Pepper 2006).

Genetic susceptibility to the development of obesity has been well documented. In this regard, it is estimated that the BMI of an individual is heritable in 40–70% of cases (Cheung and Mao 2012). Although obesity is most commonly polygenic, rare monogenic forms do exist, and the affected genes described thus far include leptin, the leptin receptor, pro‐opiomelanocortin (POMC), single‐minded 1 (SIM1), neurotrophic tyrosine kinase receptor type‐2 (NTRK2), and the melanocortin 4 receptor (Dubern and Clement 2012).

The melanocortin 4 Receptor (MC4R) is a G‐protein‐coupled receptor with the primary function of regulating food intake following the binding of the agonist alpha melanocyte stimulating hormone (*α*‐MSH). Once bound, the production of a satiety signal through the activation of the cyclic AMP (cAMP) second messenger system occurs (List and Habener 2003). Agouti‐related protein (AGRP) is an MC4R antagonist and inverse agonist and when bound produces an orexigenic signal (Govaerts et al. 2005; Chen et al. 2006).

MC4R deficiency is the most common monogenic form of obesity (Farooqi et al. 2003). Variations in the *MC4R* gene (OMIM *155541) have a population prevalence of at least one in 2000 (0.05%), are found in 0.5–1% of obese adults and are accountable for 6% of all severe cases of the disease starting in childhood (Dubern et al. 2001; Farooqi et al. 2003). These low percentages indicate that the variants have a low epidemiological significance. However, with regard to the symptomatic individual, these mutations should be considered highly significant. Individuals harboring *MC4R* mutations will preferentially transmit their mutations to their offspring with an 82% frequency, and individuals that carry pathogenic mutations have a 4.5‐fold increased risk of developing obesity when compared to noncarriers (Hinney et al. 2003; MacKenzie 2006). The frequency of *MC4R* variants is dependent on ethnicity, and differing ethnic backgrounds contribute to variability in the penetrance of *MC4R* mutations (Tao 2005; MacKenzie 2006). Recently, the role of gender has also been implicated in the penetrance of obesity‐related variants in black South African adolescents (Lombard et al. 2012).

*MC4R* mutations result in an increase in fat mass, lean mass, linear growth, extensive hyperinsulinaemia, an increase in bone mineral density, hyperphagia in early childhood and possibly binge‐eating disorder (Dubern et al. 2001; Cone 2005). The role of *MC4R* variants in the latter is however controversial (Branson et al. 2003; Lubrano‐Berthelier et al. 2006). Individuals with *MC4R* mutations also present with an elevated prevalence of the metabolic syndrome, which includes an increase in peripheral fat mass ratio, type 2 diabetes, dyslipidaemia, and hypertension (Potoczna et al. 2004). Adult subjects harboring *MC4R* mutations have an increased risk of becoming obese, and with respect to gender, it is estimated that these variants may account for up to four and 9.5 kg/m^2^ increases in BMI in males and females, respectively (Dempfle et al. 2004).

Functional defects resulting from mutations in the *MC4R* gene that are responsible for obesity include decreased or absent ligand binding; decreased cell surface receptor expression (because of intracellular retention of mutant receptors); incorrect protein folding (which results in the receptor not being released from the endoplasmic reticulum); and reduced signal transduction (Tao and Segaloff 2005; Chen et al. 2006). It has been suggested that the most common functional defect is a reduction in the constitutive activity of the receptor (Chen et al. 2006).

*MC4R* mutations have a co‐dominant pattern of inheritance, with modulation of penetrance and expressivity (Farooqi et al. 2003). Thus, homozygotes are known to be more obese than heterozygotes. However, certain *MC4R* variants appear to have a recessive pattern of inheritance, while others lead to the production of a receptor that is indistinguishable from the wild‐type (Farooqi et al. 2000).

Over 160 variants have been described for the *MC4R* gene to date (Hinney et al. 2013). Limited data regarding obesity risk alleles and their association with BMI exists in South Africa. It was therefore the aim of this study to assess the relationship between *MC4R* variants and BMI in a South African study cohort.

## Materials and Methods

### Study population

A sample cohort of the South African population was established from three groups of unrelated individuals. The first group included a random collection of individuals from the general population (*n* = 99); the second group comprised individuals that were being treated for diabetes at a tertiary hospital (*n* = 144); and the third group was established from individuals that had been assessed for obesity and its co‐morbidities by an endocrinologist (*n* = 54). The study (including informed consent forms and the proposed participant recruitment strategy) was approved by the Research Ethics Committee of the Faculty of Health Sciences of the University of Pretoria, approval numbers 102/2005, 135/2006, S103/2006 and S142/2007. The study was conducted in accordance with the Declaration of Helsinki. Age, gender, racial group, height and body weights were recorded on the day of sample collection.

### Sequence analysis of the *MC4R* gene

DNA was extracted from peripheral blood buffy coats, using a Genomic DNA Purification kit (Fermentas Life Sciences, Inc., Hanover, MD). PCR amplification of the *MC4R* gene was performed using custom designed primers MC4 EF (5′‐GCT CTG GAC TTG TGA CAT TTA C‐3′) and MC4 ER (5′‐CCA GTA CCC TAC ACG GAA GAG‐3′). The cycling conditions were as follows: 95°C/5 min; 30 cycles of 94°C/30 s, 58°C/30 s, 72°C/2 min; and a final elongation step of 72°C for 10 min. Reactions were carried out in a total volume of 50 *μ*L, which included 2 *μ*L (~100 ng/*μ*L) genomic DNA, 1.25 U GoTaq^®^ Flexi DNA Polymerase (Promega, Madison, WI), 10 *μ*L 5× buffer, 25 pmol of each primer, 200 *μ*mol/L of each dNTP, and 1.5 mmol/L MgCl_2_. Amplicons were visualized on 1% agarose gels and purified, using the DNA Clean and Concentrator ‐5 kit (Zymo Research Corporation^©^, Irvine, CA, 2005–2006).

Sequencing reactions were performed using the ABI Big Dye Terminator Cycle Sequencing kit version 3.1 (Applied Biosystems Inc., Foster City, CA) and electrophoresed by Inqaba Biotechnical Industries Pty (Ltd), South Africa. Cycling conditions were as follows: 94°C/2 min; 30 cycles of 94°C/10 s, 50°C/10 s, 60°C/4 min. Primers used included the PCR primers as externals and two newly designed internal primers: MC4 IF 5′–GCA GTG GAC AGG TAC TTT ACT ATC–3′ and MC4 IR 5′–GTC ATA ATG TTA TGG TAC TG–3′. Reactions were carried out in a total volume of 10 *μ*L, which included 0.75 *μ*L purified PCR product, 1.5 pmol primer, 2.25 *μ*L 5× dilution buffer, and 1 *μ*L ABI Prism Big Dye Terminator mix, v3.1 (Applied Biosystems). The sequencing products were cleaned using the ZR‐96 DNA Sequencing Clean‐up kit^™^ (Zymo Research Corporation^©^ Irvine, CA, 2005–2006). Products were analyzed on the Applied Biosystems/Hitachi 3130 × 1 Genetic Analyser. The *MC4R* gene was sequenced bi‐directionally in every patient in order to confirm the observed variants. These variants were confirmed by a second round of sequencing.

Electropherograms were edited using FinchTV Version 1.4.0 (Copyright^©^ 2004–2006, Geospiza Inc.) and alignments made with the MC4R sequence available from Genbank (Accession number NG_016441.1). Alignments were carried out using CLC Free Workbench Version 4.0.1 (CLC bio A/S 2005) with gap settings as follows: gap open cost: 10; gap extension cost: 1; end gap cost: as any other. The alignment was set at the “Slow (very accurate)” setting.

### Statistical analysis

Statistical analysis was performed using The Statistical Package for the Social Sciences version 22.0 (SPSS Inc., Chicago, IL). Sample distributions were tested for normality according to the Shapiro–Wilk Test. The Mann–Whitney *U*‐test was used when comparing the distribution of BMI of two groups.

## Results

### Study population

**Figure 1:**
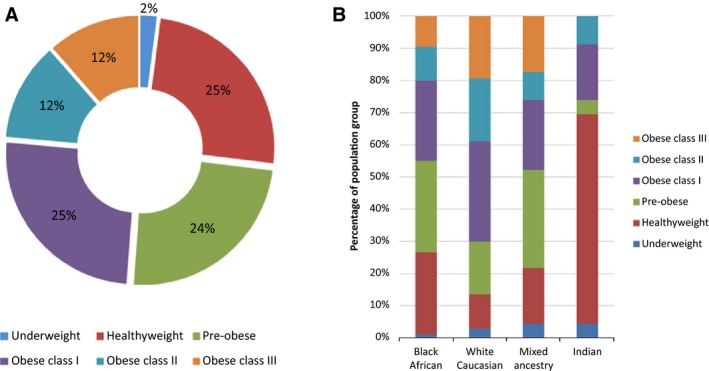
Distribution of the study cohort according to body mass index (BMI) classification. (A) Distribution according to BMI classification of the entire cohort. (B) Percentage distribution of BMI classification according to racial group. BMI classifications assigned according to the World Health Organisation (WHO): Underweight, BMI < 18.50; Healthy weight, BMI = 18.50–24.99; Pre‐obese, BMI = 25.00–29.99; Obese class I, BMI = 30.00–34.99; Obese class II, BMI = 35.00–39.99; and Obese class III, BMI ≥ 40.00.

Two hundred and ninety‐seven (*n* = 297) individuals were recruited for the study and evaluated for variants in the *MC4R* gene. The final study cohort was largely representative of the South African population, and comprised Black Africans (63.0%), White Caucasians (22.2%), Indians (7.7%), and individuals of mixed ancestry (7.1%). The study population was predominantly female (60.6%), with a mean age of 47.5 years. The mean BMI of the study cohort was 30.80 kg/m^2^ (standard deviation = 9.32). The distribution according to BMI classification (as defined by the WHO) and population group is illustrated in Figure 1. Given that the cohort was established from three different defined groups ‐ control, diabetic and obese – it was possible to recruit participants with BMIs in each of the WHO defined BMI classification groups (Fig. 1A), with nearly 75% of participants being either of healthy weight, preobese, or obese class I. This recruitment strategy did however introduce a certain level of bias, as evidenced by a non‐normal distribution of BMI (Shapiro–Wilk *P* < 0.001). With respect to racial groups (Fig. 1B), and with the exception of the Indian cohort, more than 70% of each of the recruited population groups was at least pre‐obese (BMI ≥ 25.00), while at least 45% were obese (BMI ≥ 30.00).

### Sequence analysis of the *MC4R* gene

**Figure 2:**
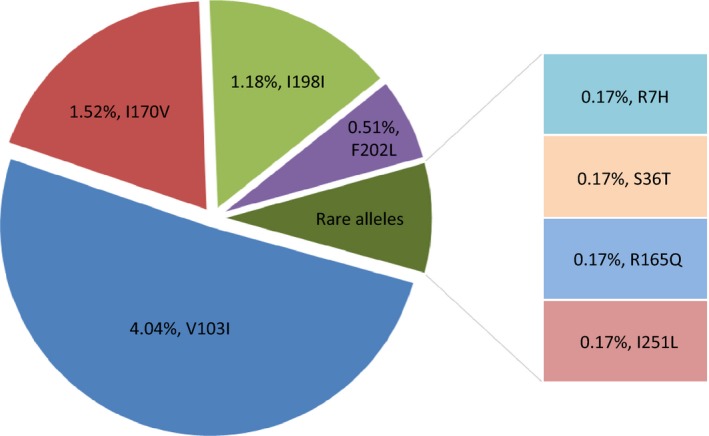
Distribution of MC4R allele frequencies in the total study cohort.

DNA sequence analysis of the entire *MC4R* gene revealed the presence of eight previously reported single nucleotide variants (SNVs, Fig. 2). The most commonly identified *MC4R* variant, V103I, was present at an allelic frequency of 4.04%. The next most frequent alleles were I170V (1.52%) and I198I variant (1.18%). The remaining five alleles were rare, and constituted an overall frequency of 1.18% (R7H, S36T, R165Q, F202L, and I251L). Five compound heterozygote individuals were identified in this study, three of whom harbored the F202L and I198I variants, while the fourth presented with the I170V and V103I alleles, and the fifth with V103I and I251L.

### Genotype‐phenotype correlation

When stratifying the study population according to racial groups and aligning this to the presence of *MC4R* variants (Table 1), it was apparent that the Black African ancestry individuals harbored the greatest degree of allelic variation when considering both presence and overall frequency of SNVs (9.89%). Only wild‐type *MC4R* variants were reported in the cohort of Indian ancestry, while the overall frequency of alleles in the White Caucasian and Mixed Ancestry cohorts was 1.07% and 1.60%, respectively. Although the Mixed Ancestry study population was limited to 23 individuals, it appeared that a greater degree of *MC4R* variation was observed in this population when compared to the White Caucasian cohort (Table 1).

**Table 1:** MC4R allelic frequencies according to this study population and from the 1000 Genomes Project

MC4R variant	This study (%)				1000 Genomes (%)^a^						
	Black African (*n* = 187)	White Caucasian (*n* = 66)	Mixed ancestry (*n* = 21)	Indian (*n* = 23)	Global	Total African (*n* = 661)	African continent^b^ (*n* = 504)	European (*n* = 503)	American (*n* = 347)	East Asian (*n* = 504)	South Asian (*n* = 489)
R7H (rs142837166)	0.27	0	0	0	–	–	–	–	–	–	–
S36T	0.27	0	0	0	–	–	–	–	–	–	–
V103I (rs2229616)	5.08	0.80	0.54	0	1.62	1.97	1.98	0.70	0.87	1.79	2.45
R165Q	0.27	0	0	0	–	–	–	–	–	–	–
I170V (rs121913560)	2.14	0	0.27	0	0.08	0.30	0.40	0	0	0	0
I198I (rs61741819)	1.34	0	0.54	0	1.28	4.46	4.27	0	0.29	0	0.31
F202L (rs138281308)	0.54	0	0.27	0	0.28	1.06	1.09	0	0	0	0
I251L (rs52820871)	0	0.27	0	0	0.26	0	0	0.90	0.43	0	0.10
Total	9.89	1.07	1.60	0	3.52	7.80	7.74	1.59	1.59	1.79	2.86

a 1000 Genomes frequency data determined via ENSEMBL Variant Effect Predictor (VEP).

b Includes only populations residing on the African continent, namely the Esan from Nigeria; Gambian in Western Division, The Gambia; Luhya in Webuye, Kenya; Menda in Sierra Leone; Yoruba in Ibadan, Nigeria.

**Figure 3:**
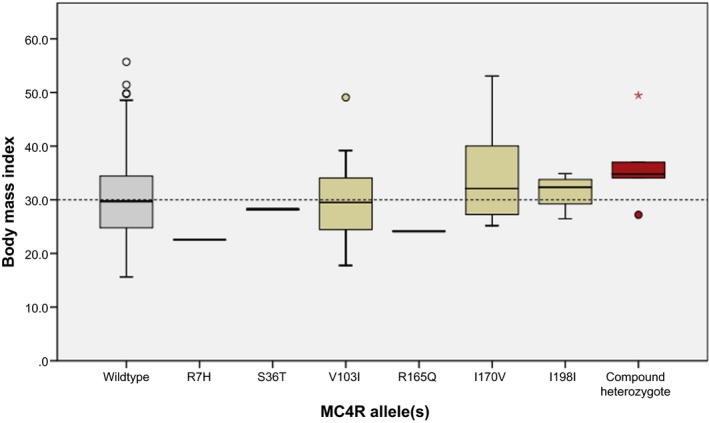
Body mass index (BMI **)** distribution of study participants harboring MC4R variants. Wildtype refers to individuals with no SNVs in MC4R; R7H through to I198I represents BMI of individuals heterozygous for these variants; compound heterozygotes refers to individuals harboring I198I and F202L (*n* = 3), V103I and I170V (*n* = 1), and V103I and I251L (*n* = 1). Since BMI data points for compound heterozygotes are represented independently and excluded from the heterozygous data sets, no box‐whiskers for individuals harboring F202L and I251L are shown. One individual, who was morbidly obese (BMI = 125.99 kg/m^2^), was excluded from the V103I data set to avoid skewing of the central tendency. Box‐whisker parameters: central tendency = median; upper and lower line of box = interquartile range; whiskers = 1.5 times the inter‐quartile range; outliers and extreme outliers are represented by open circles and asterisks, respectively.

When aligning the presence of MC4R alleles to BMI (Fig. 3), we observed that study participants heterozygous for I170V (*n* = 8) and I198I (*n* = 4) had BMIs that were mostly over 30.0 kg/m^2^, and hence were collectively classified as obese class I according to the WHO. The five individuals that were compound heterozygotes for *MC4R* variants presented with a median BMI of 34.80 kg/m^2^, but with a noticeably wide distribution ranging from 27.20 to 49.45 kg/m^2^. With particular interest in these individuals, the BMI distribution was compared to the wild‐type group by means of a Mann–Whitney *U* test, which reported no statistically significant difference (*P* = 0.069). No further groups were compared.

## Discussion

### Allelic variation of *MC4R*

Forty two of the 297 individuals (14.1%) analyzed presented with *MC4R* variants. No homozygous genotypes were observed in this study population, while five compound heterozygotes were identified, three of whom harbored the I198I and F202L alleles in combination. The fourth and fifth compound heterozygotes were both genotyped with the common V103I allele in combination with either I170V or I251L.

At 9.89%, the overall frequency of *MC4R* variants was highest in individuals of African ancestry. When compared to data derived from the 1000 Genomes Project (Table 1), this frequency appeared to be higher than the overall frequency in African populations (7.80%) and those residing on the African continent (7.74%). Moreover, the frequencies of each *MC4R* variant were also observed to differ considerably. For example, V103I was present in this study in Black Africans (5.08%) at more than twice the frequency reported in Africans globally (1.97%) and Africans living in Africa (1.98%). I170V, which was present at 2.14% in Black Africans in this study, is only present at 0.30% and 0.40% in total Africans and African residing elsewhere on the continent, respectively. The most frequent *MC4R* allele reported in Africans from the 1000 Genomes study was I198I, which was present at 4.46% in all Africans and 4.27% in Africans residing on the African continent. However, this is at least three times greater than the 1.34% reported in the Black Africans recruited in this study.

The frequencies of *MC4R* variants in White Caucasian study participants was similar to that reported in European and American populations of the 1000 Genomes Project. Interestingly, the South Asian population of the 1000 Genomes Project reported an overall frequency of 2.86% of MC4R variants, which is higher than that seen in the European (1.59%) and American (1.59%) populations. This is surprising, considering that no variants were reported in the Indian individuals participating in this study, albeit that a small sample size of *n* = 23 was recruited. Given the extensive admixture of the South African Mixed Ancestry population, it would not be reasonable to compare allelic frequencies to any populations from the 1000 Genomes Project.

### Genotype‐phenotype correlation and allelic penetrance

A great deal of controversy exists regarding the phenotypic expression of *MC4R* variants. Major contributors to this controversy are (1) the varying phenotypic penetrance of putative pathogenic variants among individuals; (2) the role of other genes in the pathogenesis of obesity; and (3) influence of the environment. An additional complicating factor is the differing approaches used by researchers when performing functional studies.

By using in silico prediction tools as a starting point (Table 2), two *MC4R* variants that are most likely to be associated with an obesity phenotype are I170V and R165Q. With respect to I170V, Tao and Segaloff originally reported that this variant had no impact on receptor function when it came to cell surface expression, agonist binding and cAMP production (Tao and Segaloff 2005). However, in 2006, Lubrano‐Berthelier and colleagues reported contradictory data, which implicated this SNV in a loss‐of‐function phenotype due to decreased cell surface expression (Lubrano‐Berthelier et al. 2006). In silico prediction of the impact of I170V via SIFT (Sorting Intolerant From Tolerant) and POLYPHEN (Polymorphism Phenotyping) reports “Tolerated (0.06)” and “Benign (0.084)” effects on the receptor. However, a “Deleterious” 0.64 score was generated from CONDEL (CONsensus DELeteriousness score). In the present study cohort, the I170V variant was identified in nine individuals, all of whom presented with BMIs of greater than 25.00 kg/m^2^ and a median BMI of 32.08 kg/m^2^ (Fig. 3). It appears therefore that I170V is indeed a contributing factor to the pathogenesis of obesity in this South Africa cohort.

**Table 2:** In silico prediction of MC4R variants reported in this study

MC4R allele	*In silico* prediction		
	SIFT	POLYPHEN	CONDEL
R7H (rs142837166)	Tolerated ‐ low confidence (0.9)	Benign (0)	Neutral (0.47)
S36T	Tolerated (0.47)	Benign (0.001)	Neutral (0.44)
V103I (rs2229616)	Tolerated (0.5)	Benign (0.279)	Neutral (0.40)
R165Q	Deleterious (0)	Possibly damaging (0.598)	Deleterious (0.63)
I170V (rs121913560)	Tolerated (0.06)	Benign (0.084)	Deleterious (0.64)
I198I (rs61741819)	NA	NA	–
F202L (rs138281308)	Tolerated (1)	Benign (0.005)	Neutral (0.43)
I251L (rs52820871)	Tolerated (1)	Benign (0.022)	Neutral (0.26)

SIFT, sorting intolerant from tolerant, data accessed via ENSEMBL Variant Effect Predictor; POLYPHEN, polymorphism phenotyping, data accessed via ENSEMBL Variant Effect Predictor; CONDEL, CONsensus DELeteriousness score (González‐Pérez and López‐Bigas 2011).

R165Q has been reported to result in a 15 to 90‐fold reduction in receptor activity when in the presence of endogenous agonists, although activity with synthetic ligands was reduced by 2 to 9‐fold (Ma et al. 2004; Xiang et al. 2006). The variant was identified in 10 out of 300 obese Pima Indians and showed a strong correlation with the development of early onset obesity (Ma et al. 2004). It also affects cell surface expression of the receptor and has generally been described as a rare allele. SIFT, POLYPHEN, and CONDEL scores of 0, 0.598, and 0.63, respectively, all point toward a likely deleterious effect of this variant. In contrast to this evidence, the single individual found to be heterozygous for R165Q in this study was of a healthy weight (BMI = 24.14 kg/m^2^).

The most frequently reported allele in this study cohort, V103I (4.04%), has been reported at similar frequencies in both obese and nonobese subjects (Dubern et al. 2001; Geller et al. 2004; Rong et al. 2006). When functionally characterized in vitro, a V103I receptor shows similarity to the wild‐type receptor and has been implicated in a gain‐of‐function phenotype that results in protection of the carrier against obesity (Geller et al. 2004). This was later supported in a large population‐based study and meta‐analysis of 29,563 individuals, where the authors report statistically significant evidence for an 18% lower risk of obesity in the presence of V103I (Young et al. 2007). Within the investigated cohort, V103I was present in individuals from all BMI classification groups (Fig. 3), confirming the lack of consensus regarding its role in obesity.

Several rare MC4R alleles were identified in this South African study. I198I was detected in seven individuals, of which three were present in compound heterozygosity with F202L. Although I198I is a synonymous variant of *MC4R*, it has been reported to be present in both obese and non‐obese individuals (Rettenbacher et al. 2007; Deliard et al. 2013). The F202L variant was identified in three individuals, and was exclusively present in combination with I198I. BMIs for these three individuals were 27.20 kg/m^2^ (preobese), 34.08 kg/m^2^ (obese class I), and 49.45 kg/m^2^ (obese class III). F202L is located in the fifth transmembrane domain of *MC4R*, and when functionally characterized in vitro, decreased basal receptor activity was observed (Tao and Segaloff 2005). In contrast, F202L was subsequently reported to have no influence on *MC4R* activity (Xiang et al. 2010), being present in both obese and nonobese individuals (Jacobson et al. 2002; Farooqi et al. 2003).

The R7H, S36T, and I251L SNVs were each reported in single cases with BMIs of 22.55, 28.23 and 34.80 kg/m^2^, respectively. R7H has been implicated in reduced *MC4R* function in vitro, due to a weakened receptor response to *α*–MSH (Lubrano‐Berthelier et al. 2003; Hinney et al. 2013). S36T was originally reported in a single obese individual and has been demonstrated in two independent studies to have no influence on MC4R activity (Larsen et al., 2005; Hughes et al., 2009). The I251L variant has been shown to have no functional implication on MC4R activity (Xiang et al. 2006) and has been reported to be present in both healthy and obese populations (Vaisse and Clement 2000; Dubern et al. 2001; Hinney et al. 2003).

Five study participants harboring two different *MC4R* variants were identified in this study. The median BMI for these compound heterozygote individuals was 34.80 kg/m^2^, which borders on an obese class II classification, and which appeared to be higher than the majority of individuals with wild‐type *MC4R* genotypes. This difference was not found to be statistically significant (*P* = 0.069). However, as evidenced in this study and in the literature, genetic variation of *MC4R* is generally observed at a very low frequency, and hence a considerably larger study cohort would be required to identify genotype–phenotype associations and differences with statistical significance.

## Concluding Remarks

Data from this investigation confirms the presence of previously reported *MC4R* variants in a South African cohort. It is widely accepted that MC4R has a part to play in the in the pathogenesis of obesity; however, the incomplete penetrance of pathogenic *MC4R* variants, the polygenic nature of obesity and the influence of environmental factors (including lifestyle) all contribute to our inability to establish a definitive molecular diagnosis for this complex disease. Since lifestyle interventions have limited success in decreasing obesity, there is an urgent need to elucidate the molecular underpinnings of this disease – on a scale similar to that described by Speliotes and colleagues, in which nearly 250,000 individuals were investigated (Speliotes et al. 2011).

## Conflict of Interest

None declared.
